# Understanding the Connection between Nanoparticle Uptake and Cancer Treatment Efficacy using Mathematical Modeling

**DOI:** 10.1038/s41598-018-25878-8

**Published:** 2018-05-24

**Authors:** Terisse A. Brocato, Eric N. Coker, Paul N. Durfee, Yu-Shen Lin, Jason Townson, Edward F. Wyckoff, Vittorio Cristini, C. Jeffrey Brinker, Zhihui Wang

**Affiliations:** 1 Department of Chemical and Biological Engineering and Center for Biomedical Engineering, University of New Mexico, Albuquerque, NM 87131 USA; 2 Advanced Materials Lab, Sandia National Laboratories, Albuquerque, NM 87106 USA; 3 Department of Internal Medicine, Division of Molecular Medicine, the University of New Mexico, Albuquerque, NM 87131 USA; 4 Center for Micro-Engineered Materials, the University of New Mexico, Albuquerque, NM 87131 USA; 5 Center for Precision Biomedicine, Brown Foundation Institute of Molecular Medicine, University of Texas Health Science Center (UTHealth) McGovern Medical School at Houston, Houston, TX 77030 USA; 6 Department of Imaging Physics, University of Texas MD Anderson Cancer Center, Houston, TX 77230 USA; 7 Department of Nanomedicine, Methodist Hospital Research Institute, Houston, TX 77030 USA; 8 Cancer Research and Treatment Center, the University of New Mexico Health Sciences Center, Albuquerque, NM 87131 USA; 9 Department of Molecular Genetics and Microbiology, the University of New Mexico Health Sciences Center, Albuquerque, NM 87131 USA; 10 Self-Assembled Materials Department, Sandia National Laboratories, Albuquerque, NM 87185 USA

## Abstract

Nanoparticles have shown great promise in improving cancer treatment efficacy while reducing toxicity and treatment side effects. Predicting the treatment outcome for nanoparticle systems by measuring nanoparticle biodistribution has been challenging due to the commonly unmatched, heterogeneous distribution of nanoparticles relative to free drug distribution. We here present a proof-of-concept study that uses mathematical modeling together with experimentation to address this challenge. Individual mice with 4T1 breast cancer were treated with either nanoparticle-delivered or free doxorubicin, with results demonstrating improved cancer kill efficacy of doxorubicin loaded nanoparticles in comparison to free doxorubicin. We then developed a mathematical theory to render model predictions from measured nanoparticle biodistribution, as determined using graphite furnace atomic absorption. Model analysis finds that treatment efficacy increased exponentially with increased nanoparticle accumulation within the tumor, emphasizing the significance of developing new ways to optimize the delivery efficiency of nanoparticles to the tumor microenvironment.

## Introduction

Cancer represents the 2nd major cause of death in the United States, and one out of eight women is estimated to develop invasive breast cancer in her lifetime^1^. Breast cancer tumors are heterogeneous, and the series of events which cause them to grow, shrink, or metastasize are complex, involving interactions with and influences from their microenvironment^2^. It is known that the heterogeneity of the breast cancer tumor greatly complicates our understanding of the disease and the development of effective treatment^2^. In fact, most tumors are heterogeneous both phenotypically and functionally^3^, resulting in variable traits among different tumors. Understanding an individual’s response based on these differing traits is essential for predicting and improving patient-specific treatment response.

Due to drug toxicity and non-specificity, a spectrum of different particles have been developed for both particle-based drug delivery and for imaging of particle distribution, including superparamagnetic iron oxide nanoparticles^4^, lipid bilayer encapsulated nanoporous silicon or mesoporous silica particles for drug/cargo delivery^5–7^, and silica based nanoparticles^8^, to name a few. Here, we discuss the use of mesoporous silica nanoparticles (MSNPs), which possess the benefits of a high cargo capacity, due to their immense internal surface area (800–1000 m^2^/g), facile surface modification to enable targeting, low toxicity, therapeutic effectiveness^9,10^, and increased circulation time, therefore increasing total tumor drug uptake^10^. As a result, the effective therapeutic drug dosage is reduced when delivered using MSNPs relative to the free drug delivery case, minimizing treatment side effects.

Using an integrated mathematical modeling and experimental approach^11^, our group has shown that nanocarrier mediated drug delivery of doxorubicin achieves equal cell kill efficacy at a dose only 20% of that of the corresponding free doxorubicin in a hepatocellular carcinoma cell model *in vitro*. In the present study, we use 50-nm diameter acetylated MSNPs, modified with polyethylene glycol (PEG) and polyethyleneimine (PEI) as first reported in Townson *et al*.^12^. These acetylated MSNPs were found to be colloidally stable and non-toxic. They were shown to have reduced non-specific binding to cell types A549, A431, Hep3b and hepatocytes *in vitro*, as well as to endothelium and white blood cells, and were observed to remain in circulation over 6 hours post injection in a chorioallantoic membrane (chicken embryo) model^12^. The increased circulation time of the acetylated MSNPs (>6 hrs) greatly improves their likelihood of entering the tumor and delivering effective drug dosages to cancer cells when compared to the circulation time of free drug (<2 hrs, t_1/2_ = 1.68 hrs)^13^.

A number of techniques have been developed to monitor the pharmacokinetics and biodistribution of nanoparticles in living animals, including optical fluorescent microscopy imaging^14–16^, ultrasound^17^, *in vivo* bioluminescence (IVIS® using luciferase)^18,19^, and PET, SPECT, and CT (as well as combinations of these techniques) using appropriate contrast agents^14,15,20–22^. However, successful prediction of treatment outcome based solely on parameters measured from the biodistribution of nanoparticles is challenging because the distribution of nanoparticles and of drug are different and heterogeneous across tissues, organs, and even whole organisms. We have previously developed a mathematical model^11,23^ that predicts the fraction of tumor killed by chemotherapeutic treatment (denoted by *f*_kill_) based on drug uptake and nanoparticle drug flux. *f*_kill_ was shown to be quadratic with respect to time, especially in the initial phase of a treatment. In the present study, we develop a mathematical theory based on this model^11,23^ in order to predict nanoparticle-based treatment efficacy using quantitative experimental data obtained from measured nanoparticle biodistribution.

Measurement of drug distribution *in vivo* is often difficult and expensive. Typically, quantitative analysis of nanoparticle biodistribution is done through organ dissection after injection of labeled nanoparticles, followed by imaging, using methods such as transmission electron microscopy or optical microscopy, or by elemental analysis using inductively coupled plasma-atomic emission^24^. Nanoparticle distribution can also be measured *in vivo* using magnetic resonance imaging and magnetic particle imaging^24^. Here, capitalizing on the low natural abundance of elemental silicon in mammals, we performed Si elemental analysis of the major organs in order to measure MSNP concentrations, and then compared these quantified MSNP accumulation values to changes in tumor volume. By using our theory to link measured tumor growth with nanoparticle distribution and concentration, and considering the effects of vasculature and diffusion characteristics, we were able to successfully predict the *in vivo* therapeutic efficacy of MSNP-delivered doxorubicin to 4T1 breast cancer tumors in mice.

## Results and Discussion

Cancer stage at the time of diagnosis is demonstrated to be an important predictor of morbidity and survival^25^. Five-year survival rates for breast cancer are 99% when diagnosed pre-metastasis, but with a significant reduction to only 25% five-year survival rate when the tumor has metastatized^25^. In order to better understand the difficulties of effective theraputic treatment when patients are diagnosed at a later stage, our experiments focused on treating tumors that were relatively large with distant metastases prior to the start of a treament. Accordingly, we implemented a 4T1 cell line experiment in BALB/c mice, in order to study stage IV human breast cancer with metastasis in the presence of an active immune system.

**Figure 1:**
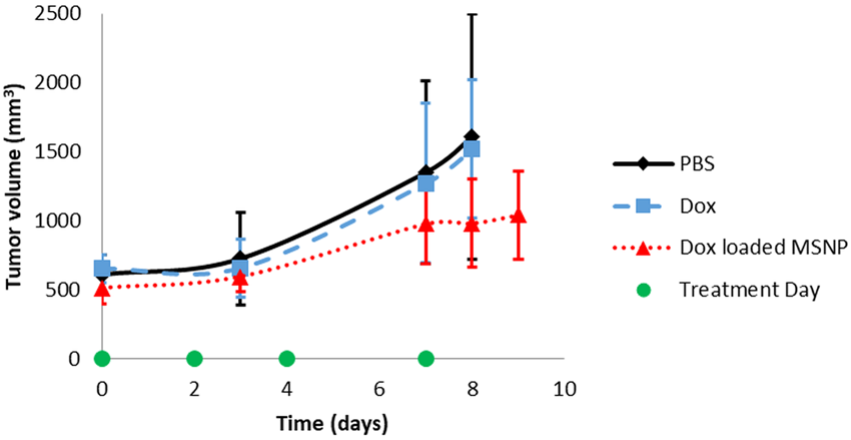
Average tumor volume measurements. Three treatment groups (7 mice/group, with standard deviation shown in error bars): PBS (control), free doxorubicin (Dox), and 50 nm MSNPs loaded with Dox. Each group’s average tumor size is shown above. Measurements were taken on days 0, 3, 7, 8, and 9. Treatments were given on days 0, 2, 4, and 7. Individual mice data are shown in Supplementary Fig. S1.

Treatment efficacy between MSNPs loaded with doxorubicin (Dox) and free Dox was compared at the same dosage; phosphate buffered saline (PBS) was used as a control in a third treatment group. Tumor size measurements in mm^3^ are shown in Fig. 1 for three groups of seven mice each treated using PBS (control), free Dox, or MSNPs loaded with Dox. Due to this treatment being adminstered at a later stage of breast cancer, it can be observed that free Dox treatment is not effective at a late stage; but the nanoparticle treatment shows some efficacy (see Supplementary Fig. S1 for each individual measurement). However, we note that statistically significant difference in tumor response between different treatment groups was not identified. This warrants additional experiments on a larger scale with more data points to demonstrate the treatment efficacy for this particular nanoparticle.

**Figure 2:**
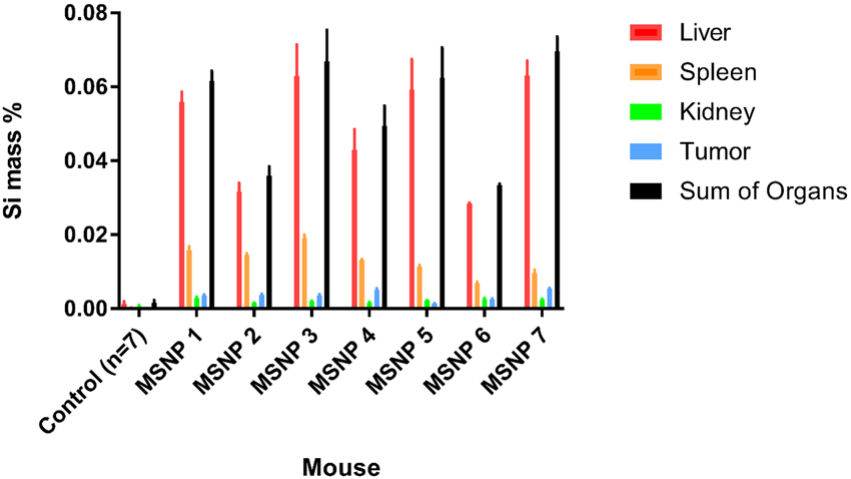
MSNP deposition (Si mass %) in liver, spleen, kidneys, tumor, sum of organs, and average Si concentration naturally in tissues (control group) taken post-sacrifice (day 9). Data were obtained using GFAA spectrophotometry. Error bars are calculated based on the standard additions method used to calculate standard deviation of Si concentrations.

*In vivo* biodistribution of nanoparticles based on size, shape, composition, and surface characteristics is still not well understood, as are the details of nanoparticle removal from circulation by the reticuloendothelial system^26^. To gain a better understanding of these important parameters, we used graphite furnace atomic absorption (GFAA) to measure silicon (Si) concentration and distribution within tissues of interest^27^. The Si concentration in the control (PBS) group was used as a baseline for the background Si concentration which occurs naturally in tissues (Si has important biofunctionality, and thus is found in trace quantities in many tissues). As expected, we observed that the amount of naturally occurring Si in the control tissues was low relative to the signal of MSNP in treated tissues (Fig. 2, control). Figure 2 shows measured values of elemental Si determined by GFAA, presented as the mass percentage of Si in the corresponding tissue being tested in mouse organs from the control and Dox loaded MSNP treatment groups after 9 days of treatment and sacrifice. The tissues tested were tumor, kidney, liver, spleen, as well as a measured sum of all organs. The sum of all organs tested corresponded to ~2–4.4% of the total injected Si dose, as shown in Supplementary Fig. S2.

**Figure 3:**
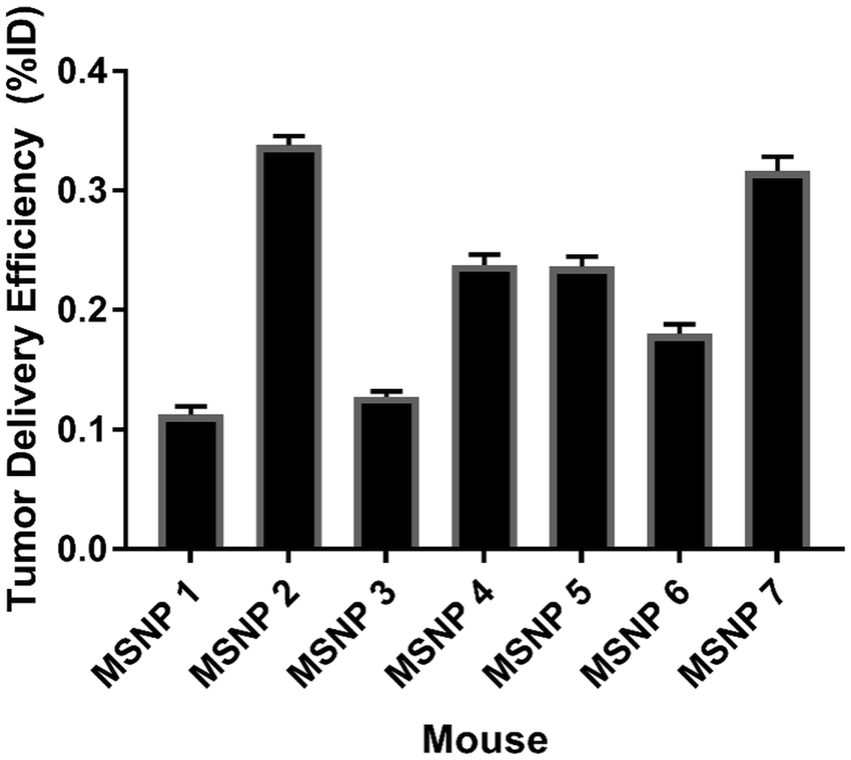
Tumor delivery efficiency (%ID). Data were obtained using GFAA spectroscopy. Naturally occuring Si (measured by testing control tumors in mice not exposed to MSNPs) was subtracted from absolute Si mass % in tumor. Standard deviation is shown in error bars (*n* = 7 for control and MSNP groups). Delivery efficiency calculation is described in Methods in section “Graphite Furnace Atomic Absorption Spectrophotometry.”

Mice, even within the same treatment group, demonstrated a wide range of *f*_kill_ responses, indicated by the large standard deviation seen in tumor volumes in Fig. 1 (error bars). Moreover, the concentrations of Si deposited in the liver, spleen, kidney, and tumor were shown to vary without correlation between uptake in the tissues measured in our study (Fig. 2). For example, mice that had greater MSNP uptake in the liver did not have greater or lesser uptake in the tumor, see Fig. 3 for tumor delivery efficiency (%ID), and mice with less MSNP uptake in the tumor did not have less or more uptake in other organs. From our experiments here, we find that the MSNP delivery efficiency to the tumor is about 0.22%ID on average. Based on an extensive review of papers over the past ten years, Wilhelm *et al*. reports 0.7%ID as being the average tumor delivery efficacy using a multitude of particles, cancer types, and measurement methods, and mentions that, “it is possible that the amount of nanoparticles reaching cancer cells and their subcellular compartments *in vivo* is much less than 0.7%ID because nanoparticles need to cross the tumour extracellular matrix to reach the cancer cells”^28^. Hence, our data seems well consistent with Welhelm *et al*.’s paper. One paper used elemental analysis, ICP-AES, to measure silica nanoparticle concentration, of which the %ID was determined to be 0.29, 1.6, and 10.8%ID^29^, most of the quantifications used to calculate the average of 0.7%ID in a tumor used PET scans as a method of quantification^16,21,30,31^. Thus, the biodistribution of nanoparticles was observed to show significant variability, even amongst similar mice under the same treatment protocol. Blood volume fraction (i.e., the volume of the tumor occupied by blood vessels) was previously shown to be an important factor in predicting *f*_kill_ in human colorectal cancer metastasized to the liver^32^.

**Figure 4:**
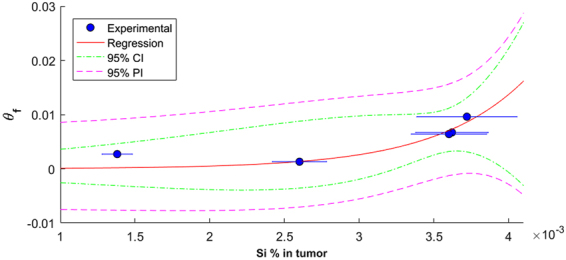
Si concentration and *f*_kill_ coefficient (*θ*_*f*_) determination. *θ*_*f*_, explained in Eq. 4, is predicted based on Si absolute mass percentage in tumor, as measured using graphite furnace atomic absorption (GFAA). *R*^2^ = 0.817, *p*_.05_ = 0.0007 for quadratic *f*_kill_ coefficients calculated after 9 days of treatment and sacrifice. This was determined by the best fit between *f*_kill_ and Si concentration using an exponential fit (which represents the best fitting function among many we have tested, including linear and nonlinear functions, e.g., quadratic, sigmoidal, the Michaelis-Menton, and the Hill function). Experimental mouse tumor *f*_kill_ was determined using Eq. 2 for mice treated with MSNPs. Model mouse tumor *f*_kill_ was determined by optimizing values for *t*_0_ (days in integer values), described in Eq. 4. Standard deviation for measured Si % in each mouse’s tumor treated with MSNPs is shown for experimental values on the x-axis.

Many current nanoparticles have achieved the capability to release drugs at a nearly constant, sustained rate for a period of days, weeks, or even months^33–35^, although this rate may be dependent on other physiochemical properties of the particles, such as the surface chemistry and pore size^36^. *In vivo*, this nearly constant drug release rate results in an approximately unchanged rate of change of drug flux (denoted by *F*) across blood vessels. By further assuming a linear drug uptake by cancer cells, we have devleoped a special form of *f*_kill_ for predicting treatment efficacy for nanoparticle-based drug delivery systems^23^. We found that tumor response *in vivo* to Dox loaded nanoparticles occurs quadratically over time, at least for the first several days (${f}_{{\rm{kill}}}={\theta }_{f}\cdot {t}^{2}$, i.e., Eq. 4, where *θ*_*f*_ is the tumor *f*_kill_ coefficient; also see Methods), and have further validated this model using experiments on a breast cancer mouse model. Accordingly, we used this quadratic tumor response model to link the MSNP deposition with measured changes in tumor volume (relative to control). The quadratic tumor response coefficient (i.e., *f*_kill_ coefficient: *θ*_*f*_) was determined to have an exponential relationship with MSNP deposition in the tumor tissue (Fig. 4); note that we simply applied a phenomenological approach to identification of the relationship between *θ*_*f*_ and Si deposition. *θ*_*f*_ was found to be predictable based on tumor silicon content with 95% confidence (*R*^2^ = 0.817, *p*_.05_ = 0.0007) based on the quadratic curve for *f*_kill_ described in Eq. 4. This indicates that increasing chemotherapy drug delivery, using a MSNP transport vector, results in an exponentially greater rate of tumor kill. Values for comparison are shown in Table 1, along with statistics that indicate that most model values are statistically significant at 95% confidence except for MSNP 5. MSNP 4 and 7 did not show measurable response to treatment until day 7, resulting in only two data points; therefore, *p*-values for *θ*_*f*_ could not be determined due to an insufficient number of points. As such, these two mice were removed from further analysis. Together, this indicates that MSNP uptake is an important factor in determining tumor treatment efficacy. However, more tumor measurements and/or a longer experiment would be beneficial to validate statistical significance with the model predictions.

**Table 1:** Tumor *f*_kill_ coefficients (*θ*_*f*_), as described by Eq. 4, show a similar order to Si deposition values.

Mouse	Si % in tumor	*t*_0_ (time tumor begins to respond to treatment in days)	*θ*_*f*_ (quadratic response coefficient)	*p*-value	*R*^2^ (quadratic)
MSNP 4	0.00513	—	0.4621	—	—
MSNP 7	0.00535	—	0.3068	—	—
MSNP 2	0.00372	0	0.0096	0.0030	0.9648
MSNP 3	0.00362	0	0.0067	0.0050	0.9499
MSNP 1	0.00360	0	0.0064	0.0110	0.9155
MSNP 5	0.00138	0	0.0027	0.1990	0.4728
MSNP 6	0.00260	0	0.0013	0.0060	0.9409

The Si deposition values are also shown in Fig. 2 (MSNP deposition) and in Fig. 4 (R^2^, *θ*_*f*_, *t*_0_), showing the exponential relation to the tumor *f*_kill_ coefficient calculated using Eq. 4. *p*-values and *R*^2^ are calculated with respect to quadratic curves described in Eq. 4.

We then performed correlation analysis to compare model results (computed as ${f}_{{\rm{kill}}}=0.000172\cdot {e}^{1664.39\cdot Si}\cdot {t}^{2}$, where *Si* is tumor Si mass %) and the corresponding time course MSNP experimental data as shown in Fig. 1; also see Fig. S3. We obtained correlation coefficient *r* = 0.89 and *p* < 0.001, and thus consider the model to be acceptable in predicting MSNP-based treatment outcome with absolute Si mass percent as input.

Cancer treatment may be improved by using MSNPs as chemotherapy delivery vessels, which allow for an increased effective drug dose to be delivered to the tumor site due to drug sequestration within the particles during transit to the site, resulting in reduced uptake by the reticuloendothelial system and longer circulation time^37^. We have provided further insight into this drug delivery system using MSNPs in a murine 4T1 *in vivo* tumor model combined with a mathematical modeling description of drug efficacy. In particular, our mathematical model demonstrates that an increase in MSNPs delivered to the tumor exponentially increases the cell kill at early times in the treatment, leading to improvements in overall treatment outcome. The major hurdle is thus increasing tumor MSNP delivery over current methods, as any increase in delivery to the tumor is expected to significantly improve treatment efficiency. In this perspective, active tumor targeted delivery of chemotherapy via targeting ligand modified nanoparticles^5,38^ is expected to enhance treatment efficacy. We also clarify that, as described in our prior work^23^, the drug concentration used to derive the *f*_kill_ model is the concentration at the blood vessel wall, which is assumed to be a constant and is averaged over the duration of treatment, i.e., the actual drug concentration in the tissue which reaches the tumor was not measured previously. In the present work, we have obtained an actual measurement for the estimated drug concentration in the tissue through measuring the carrier (MSNPs) loaded with Dox in the tumor and MSNP uptake in organs.

Morover, if the quadratic *f*_kill_ coefficient could be determined early on, the treatment efficacy could be predicted by our mathematical model. It has been demonstrated that tumor exponential growth rate constants were correlated to patient survival^39^. Here, we demonstrate that, due to external stresses and regression in tumor size due to treatment, the quadratic coefficient following treatment is predictive of treatme006Et uptake and therefore treatment efficacy. Note that *θ*_*f*_ is a combination of parameters, inlcuding drug flux across blood vessels, cell death due to accumulated drug, and initial tumor volume (see^23^ for details). This implies that *θ*_*f*_ may have a nonlinear relationshiop with Si content, which has been confirmed in this study where an exponential relation was found. In future efforts, a better understanding of the biodistribution of nanoparticle and Si across organs examined on a larger dataset will allow us to have a more adaptive use of the model presented here. Additionally, further model validation against information obtained from non-invasive imaging modalities such as MRI, PET/CT^40^ will help to quantify nanoparticle-based treatment outcome without the need to sacrifice animals. This important next step will also progress the model towards a more clinical functionality, where it may be implemented as a predictive tool without the need for invasive diagnostic procedures.

## Materials and Methods

### Mathematical modeling

We recently developed a series of mathematical models in closed form for predicting tumor response to treatment based on time- and space-dependent drug diffusion and perfusion properties^11,23,32,41–47^. A generalized model presented in^23^ can provide predictions of outcome for both conventional chemotherapy with a specific dosing and timing regimen and nanoparticle-based treatment. *f*_kill_ (i.e., the fraction of tumor killed by treatment) is defined as:

1 $${f}_{{\rm{kill}}}=1-\frac{{V}_{i}(t)}{{V}_{{\rm{C}}}(t)},$$

where *V* is tumor volume at time *t*, *i* indicates drug treatment group (Dox or Dox loaded MSNPs), and C indicates the control group. Normalizing tumor volume at a given time *t* to initial volume, we have

2 $${f}_{{\rm{kill}}}=1-\frac{{V}_{i}(t)/{V}_{i}({t}_{0})}{{V}_{{\rm{C}}}(t)/{V}_{{\rm{C}}}({t}_{0})},$$

where *t*_0_ is determined to be the initial day when treatment was started; in our analysis here, *i* simply indicates either free DOX or MSNP treatment group.

Assuming that drug-loaded nanoparticles can accumulate within tumors and continuously release drugs at a nearly constant rate over a certain time interval (especially at the initial phase of a treatment), we derived a special form of *f*_kill_^23^:

3 $${f}_{{\rm{kill}}}=\frac{F\cdot {{\boldsymbol{\lambda }}}_{{\rm{k}}}}{2{V}_{{\rm{T}},0}}{t}^{2},$$

where *F* is the flux of drug across blood vessel walls, ***λ***_k_ is the death rate of tumor cells, *V*_T,0_ is the tumor volume when tumor begins to respond to treatment (positive *f*_kill_), and *t* is time. Note that there is another key assumption we made to the original *f*_kill_ model, which is composed of a system of differential equations, in order to develop this simplified form: a drug administered as bolus at a certain dose level has the same effect as the same total amount of drug administered over several months at a constant, smaller dose level. That is, this model functions for a number of situations, including (1) single drug injection at the beginning, (2) multiple drug injections over the course of treatment, and (3) continuous drug administration. Regardless of how we administer the drug, the treatment system can be modeled as a continuous drug delivery system. This assumption has been validated previously *in vivo* and in patients across different types of cancer^23,32^.

Parameters for best model fits to experimental data (determined by Eq. 2) with the model’s predictions from Eq. 3 are derived from:

4 $${f}_{{\rm{kill}}}={\theta }_{f}\cdot {t}^{2},$$

where *θ*_*f*_ is the tumor *f*_kill_ coefficient $({\theta }_{f}=\frac{F\cdot {{\boldsymbol{\lambda }}}_{{\rm{k}}}}{2{V}_{{\rm{T}},{\rm{0}}}})$. From our experimental analysis, we obtain:

5 $${\theta }_{f}=A\cdot {e}^{B\cdot Si},$$

where coefficients *A* and *B* are fit to determine best values for prediction between Si absolute mass percentage and the model output, *θ*_*f*_. *A* and *B* are tumor and drug specific coefficients specific to 4T1 breast cancer given treatment when tumors are ~500 mm^3^ when beginning treatment. *A* and *B* are the same for all 5 mice given the treatment described under the experimental description used to describe the relationship between Si mass % deposited in 4T1 breast tumors and *θ*_*f*_, shown in Fig. 4.

### Experiment description

#### Accordance statement

Mouse experiments were performed using protocol approved by the UNM Office of Animal Care Compliance. All methods were carried out in accordance with relevant guidelines and regulations. Six- to eight-week-old female BALB/c female mice were given subcutaneous injections of 5 × 10^5^ 4T1 (ATCC® CRL2539™) cells into the right flank. Tumors were grown for two weeks before treatment initiation. Average tumor volumes, calculated from diameters measured externally with calipers, are shown in Fig. 1. Mice were randomly divided into three treatment groups (7 mice/group, 21 mice total): control (PBS), free doxorubicin 1 mg/kg per treatment, 1 mg acetylated MSNPs (50 nm, 2.5 nm pores) loaded with doxorubicin (equivalent 1 mg/kg doxorubicin per treatment). Acetylated MSNPs were made using the protocol described in Townson *et al*.^12^, and loaded with doxorubicin using the drug loading protocol for water soluble doxorubicin in Lin *et al*.^48^. Treatment was given starting 2 weeks after tumor cell injections. Treatment days were as follows (*t* = 0 is 2 weeks following tumor injection): 0, 2, 4, and 7. All mice were sacrificed on day 9. Tumor measurements were taken on days: 0, 3, 7, 8, and 9. Tissues were excised and fixed in 4% formaldehyde diluted in PBS, and then Si contents were determined using graphite furnace atomic absorption spectrometry as described below. Statistical analysis was conducted using Matlab, Excel, and Graphpad Prism.

### Graphite Furnace Atomic Absorption Spectrophotometry (GFAA)

Analysis of Si concentration in MSNP (mesoporous silica nanoparticle) and control (PBS) treated mice tissue was tested using a THGA graphite furnace on a PinAAcle 900 T Atomic Absorption Spectrophotometer (Perkin Elmer, USA). Tissues tested were tumor, kidney, spleen, and liver digested using aqueous tetramethylammonium hydroxide. Absolute mass percentages of silicon in the specific tissue were measured using the standard additions method, commonly used for samples prepared within a matrix that has a strong influence on the analyte signal (the matrix is defined as anything present in the solution that is not the analyte). In the standard additions method, tissues are spiked with differing, known concentrations of Si in order to determine the Si mass % in the tissue measured by extrapolation of the obtained curve of Si signal versus addition to zero addition; a unique signal vs. addition curve is generated for each tissue sample. Tumor (or organ) delivery efficiency, %ID, was determined by using BALB/c standard organ values from Tsai *et al*.^49^ to estimate total organ Si mass deposited. Total Si delivered was calculated based on Si percentage (39.36% of nanoparticle mass) added during synthesis using the protocol in Townson *el al*.^12^. Tumor delivery efficiency, %ID, was determined using Si absolute mass percentage in the average control mice each respective organ subtracted from that of the MSNP treated mouse and dividing it by the total administered dose over the treatment duration. The delivery efficiency was measured based on the mass of Si measured in each respective organs divided by the total injected mass of Si (in the form of MSNPs). See Supplementary Information for more details on GFAA methods.

### Data availability

All data generated or analyzed during this study are included in this published article and its Supplementary Information files.

## Electronic supplementary material

Supplementary Information
