# Supplementary material for: Understanding the Connection between Nanoparticle Uptake and Cancer Treatment Efficacy using Mathematical Modeling
Source: Sci Rep. 2018 May 24;8:7538. doi: 10.1038/s41598-018-25878-8 (PMC5967303; doi:10.1038/s41598-018-25878-8)
Supplement: Supplementary file 1 — Supplementary Information [file 41598_2018_25878_MOESM1_ESM.docx]

**SUPPLEMENTARY INFORMATION**

Understanding the Connection between Nanoparticle Uptake and Cancer Treatment Efficacy using Mathematical Modeling

Terisse A. Brocato, Eric N. Coker, Paul N. Durfee, Yu-Shen Lin, Jason Townson, Edward F. Wyckoff, Vittorio Cristini, C. Jeffrey Brinker, and Zhihui Wang

Graphite Furnace Atomic Absorbance (GFAA) Spectrophotometry Method Optimization- While keeping the pyrolysis temperature constant, atomization temperature was tested through a temperature range of 2300-2500°C in order to determine which temperature gave the optimal peak area and lowest standard deviation. It was determined that atomization temperature did not seem to play a role in maximizing peak area. While atomization temperature was kept constant, pyrolysis temperature was tested from 1100-1425°C. The greatest peak area was shown to be 1325°C, but only offers 10°C temperature control incrimination, therefore 1320°C was chosen as the method’s pyrolysis temperature.

**Table S1.** Graphite Furnace Atomic Absorbance Spectrophotometry Atomization Optimization-200ppb Si standard was tested at different atomization temperatures while keeping pyrolysis temperature at 1400°C. 2400°C was chosen for atomization temperature, as atomization temperature seemed to exhibit negligible influence on measurement quality within this temperature range.

| Atomization temperature (°C) | Blank Corrected Absorbance | Standard Deviation (n=4) |
| --- | --- | --- |
| 2300 | 0.20699 | 0.00890 |
| 2325 | 0.20820 | 0.00531 |
| 2350 | 0.20729 | 0.00334 |
| 2375 | 0.19672 | 0.00639 |
| 2400 | 0.19847 | 0.00814 |
| 2425 | 0.19582 | 0.01071 |
| 2450 | 0.20004 | 0.00234 |
| 2475 | 0.19933 | 0.00291 |
| 2500 | 0.19444 | 0.00431 |

**Table S2.** Graphite Furnace Atomic Absorbance (GFAA) Spectrophotometry Pyrolysis Optimization-200ppb Si standard was tested at different pyrolysis temperatures while keeping atomization temperature at 2400°C. 1320°C was chosen for pyrolysis temperature for all measurements, as optimized based on largest absorption readings, minimization of standard deviations, and machine temperature control tolerances.

| Pyrolysis Temperature (°C) | Blank Corrected Absorbance | Standard Deviation (n=4) |
| --- | --- | --- |
| 1100 | 0.14571 | 0.01635 |
| 1125 | 0.13450 | 0.02223 |
| 1150 | 0.14935 | 0.01650 |
| 1175 | 0.14581 | 0.02227 |
| 1200 | 0.15471 | 0.01168 |
| 1225 | 0.17393 | 0.02454 |
| 1250 | 0.19250 | 0.02122 |
| 1275 | 0.20091 | 0.02217 |
| 1300 | 0.20267 | 0.01484 |
| 1325 | 0.21009 | 0.00925 |
| 1350 | 0.20224 | 0.01237 |
| 1375 | 0.19042 | 0.00242 |
| 1400 | 0.20013 | 0.01467 |
| 1425 | 0.19025 | 0.00780 |

Graphite Furnace Solutions and Tube Coatings- Argon Ultra High Purity 99.999% (Matheson Tri-Gas UN1006) was used to purge the furnace chamber during and between runs (except during signal measurement). Tissue was digested using 250 g/L tetramethylammonium hydroxide (TMAOH, Sigma-Aldrich) in order to create a homogeneous solution for pipetting into GFAA (see Supplementary ­­­­­­­­Table S3). GFAA determination of Si concentration used 4 µl of 2.5 ug/µl Pd as a matrix modifier, under temperature conditions optimized for pyrolysis and atomization (data shown in Tables S1 and S2). A permanent matrix of ZrCl_4_ was used on the furnace tube to improve furnace lifetime, 40 µl of 500 mg/L Zr solution was used every 300 cycles. Si absolute mass percentages were determined using the standard additions method with solutions containing 20µl of: 0, 100, 200 ppb Si standard solution for liver, spleen, and kidney tissue, and 0, 100, 150, 250 ppb Si standard solution (1000 mg/L Si solution, PerkinElmer PE# N9303799) for tumor tissue. *N* = 4 for each concentration in standard additions curves. Each curve was required to have a *R*^2^ greater than 0.990.

**Table S3.** Tissue dissolved in TMAOH, concentrations and dilution were implemented to minimize furnace exposure to TMAOH (which reduces tube lifetime).

| Tissue Tested | Tissue in TMAOH (g/ml) | Dilution TMAOH/tissue digest in water (ul/ml) |
| --- | --- | --- |
| NP kidney | 0.35 | 35 |
| control kidney | 0.35 | 35 |
| NP spleen | 0.35 | 2.5 |
| control spleen | 0.35 | 25 |
| NP liver | 0.15 | 2.5 |
| control liver | 0.15 | 25 |
| NP tumor | 0.176 | 50 |
| control tumor | 0.645 | 50 |

**Figure S1**

**A**

**B**

**C**

**Figure S1. Tumor volume measurements of all the mice.** Three treatment groups (7 mice/group): (A) PBS (control), (B) free doxorubicin (Dox), and (C) 50nm MSNPs loaded with Dox. Measurements were taken on days 0, 3, 7, 8, and 9. Treatments were given on days 0, 2, 4, and 7.

**Figure S2**

**Figure S2. Delivery efficiency (%ID) in liver, spleen, kidneys, tumor, and sum of organs.** Data were obtained using GFAA spectroscopy. Absolute Si mass % in organs were subtracted from naturally occuring Si measured by testing control mice not exposed to MSNPs.

**Figure S3**

**Figure S3. Time plot with predicted *f*_kill_ model (**$f_{\text{kill}}=0.000172\cdot e^{1664.39 \cdot Si}\cdot t^{2}$**)** **on y-axis.**
